# Highlights from the 4th PALOP-AORTIC Conference on Cancer, 29–31 July 2020, Luanda, Angola

**DOI:** 10.3332/ecancer.2020.1108

**Published:** 2020-09-21

**Authors:** Lúcio Lara Santos, Fernando Miguel, Satish Túlsidas, Hirondina Borges Spencer, Belmira Rodrigues, Lygia Vieira Lopes, Helga Freitas

**Affiliations:** 1 Experimental Pathology and Therapeutics Research Group, and Surgical Oncology Department, Portuguese Institute of Oncology, Rua Dr António Bernardino de Almeida 4200-072, Porto, Portugal; 2 ONCOCIR—Education and Care in Oncology—Lusophone and Africa, Rua Dr António Bernardino de Almeida 4200-072, Porto, Portugal; 3 Angolan Institute Against Cancer, Rua Amílcar Cabral, Luanda, Angola; 4 Medical Oncology Service, Maputo Central Hospital, 1653 Av Eduardo Mondlane, Maputo, Mozambique; 5 Medical Oncology Service, Agostinho Neto Hospital, Rua Borjona de Freitas, Plateau, Praia 112, Cape Verde; 6 AORTIC Managing Director, AORTIC - PO Box 186, Rondebosch 7701, South Africa; 7 Cancer Unit, Sagrada Esperança Clinic, Av Murtala Mohammed 298, Luanda, Angola; 8 Director of Public Health of Angola, Ministry of Health of Angola, Largo Josina Machel, Luanda, Angola

**Keywords:** Lusophone African countries, oncology, AORTIC, PALOP, conference

## Abstract

The 4th *Países Africanos de Língua Oficial Portuguesa* (PALOP)-African Organisation for Research and Training in Cancer (AORTIC) Conference on Cancer was held in July 2020 in Luanda, Angola, under the theme: ‘Training to better care’. It was hosted by the Ministry of Health of Angola and AORTIC. It was held virtually using an online platform. The PALOP organisation comprises Lusophone African countries. The conference brought together 360 delegates from 12 countries. Key themes covered during the conference included: Instruments for Proficient Cancer Control in PALOP, oncology education and training in PALOP, CanScreen5—International Agency for Research on Cancer platform to improve quality in tracking cancer, International Gynecologic Cancer Society—Global Curriculum and Mentorship Programme, Oncology Training/Intervention—Support Programmes, Telepathology and Cancer: Challenges and Opportunities, Cancer Burden in PALOP region and Sub-Saharan Africa, Breast Cancer—The current situation in PALOP and The African Breast Cancer Coalition—Disparities in outcomes study in PALOP Countries (ABC-DO-PALOP) study: a proposal. It has been demonstrated that the collaboration and exchange of experiences between African countries and amongst PALOP, in particular, are crucial, whether in the organisation of population-based cancer registries, in the realization of national oncology plans, in the creation of therapeutic recommendations and in strengthening capacities in radiotherapy, amongst other important topics in oncology. The PALOP oncology school will be a fundamental training tool to be administered for better care for cancer patients.

## Introduction

We are experiencing a health emergency resulting from the worldwide SARS-CoV-2 infection that has caused many fatalities and impacted on healthcare resources. However, endemic diseases and chronic diseases continue to afflict African Portuguese-speaking countries. Oncological diseases in Africa are on the rise, and it is estimated that, in 2040, more than 2 million new cases will occur. In the African Portuguese-speaking populations, there will be approximately 100,000 cases, an expressive increase [1]. It is essential that countries prepare adequately to face these numbers.

The Portuguese-speaking countries in Africa include Angola, Mozambique, Guinea-Bissau, Cape Verde, São Tomé and Principe, which form an interstate organisation known as *Países Africanos de Língua Oficial Portuguesa* (PALOP). In order to better coordinate actions to control cancer, the PALOP gathered at the 9th African Organisation for Research and Training in Cancer (AORTIC)’s International Conference in Durban, South Africa, and pledged to work together. From this moment, the PALOP Group of Oncology was created [2].

The first cancer meeting organised by PALOP occurred in October 2014 in Luanda, Angola. In 2016, the second cancer conference was organised in Maputo, Mozambique, and the third entitled ‘quality in cancer care, optimisation of cancer units, cancer education and training’ took place in 2018 in Praia, Cape Verde [2]. These conferences had the scientific support of the AORTIC and are dedicated to the improvement of cancer care in PALOP.

Angola was designated as the country responsible for organising the 4th PALOP conference. In view of the pandemic, AORTIC and the PALOP Group of Oncology made the decision to hold a virtual conference from 29 to 31 July 2020.

## Conference summary

**Figure 1.**
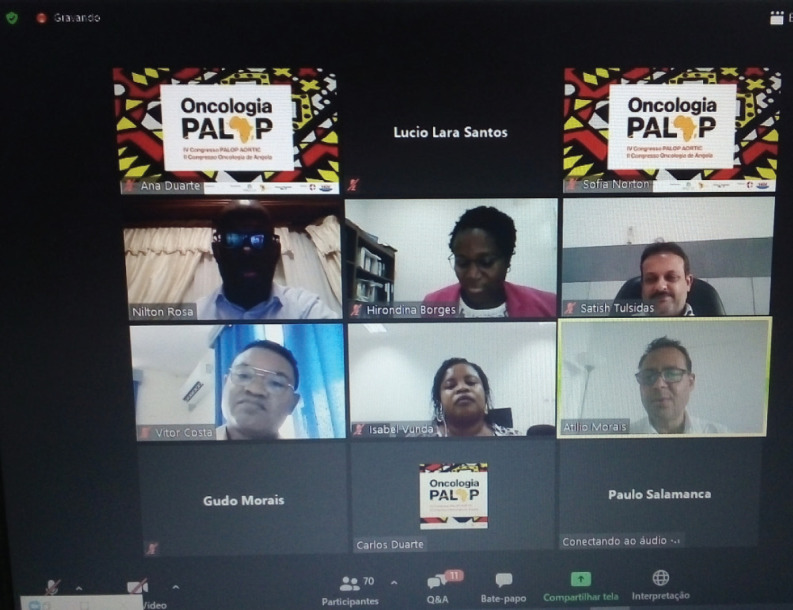
IV PALOP-AORTIC Conference (Virtual). Oncology education and training in PALOP session. (Photographed participants authorised their publication.)

The PALOP conference, held virtually online, brought together 360 delegates from Angola, Mozambique, Cape Verde, Nigeria, South Africa, Zambia, Tanzania, Portugal, Brazil, France, UK and the USA. It brought together clinicians, academics, researchers, Ministry of Health officials and representatives from cancer institutions such as International Agency for Research on Cancer (IARC), NIH, International Gynecologic Cancer Society (IGCS), Portuguese Society of Oncology, ECHO/MD Anderson and Calouste Gulbenkian Foundation, to share knowledge and adopt best practice in oncology. The average number of participants in the sessions was 82 (64–88 participants) (Figure 1).

**Figure 2.**
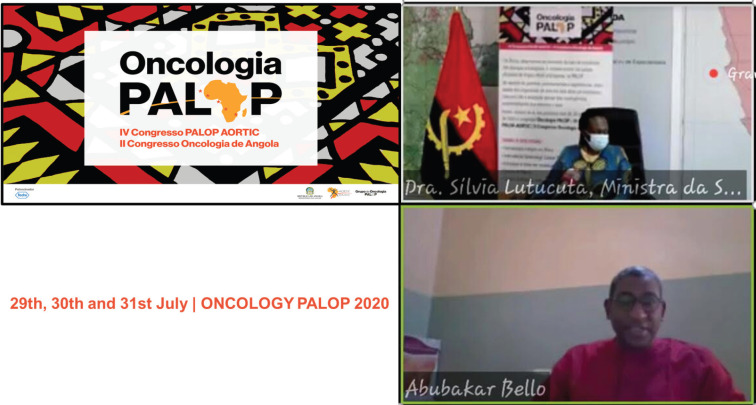
Open session. Silvia Lutucuta, Minister of Health of Angola and Abubakar Bello, President of AORTIC. (Photographed participants authorised their publication.)

ROCHE® sponsored the cost of the conference in accordance with their African Project to strengthen healthcare systems and cancer care. Each session had two interpreters to translate, simultaneously, from Portuguese to English or from English to Portuguese depending on the language used by the speaker during their presentation. The opening address of the conference was given by the President of AORTIC Prof. Abubakar Bello and the Minister of Health of Angola Dr Silvia Lutucuta (Figure 2). The Conference closure was carried out by the PALOP representative on the AORTIC Council Prof. Cesaltina Lorenzoni and the National Director of Public Health of Angola, Dr Helga Freitas.

The scientific programme included a variety of plenary sessions (25 presentations plus panel discussions) and discussion of clinical cases. Presentations were given across the oncological continuum of care. Strategies help to change in risky behaviour, increase in vaccination against hepatitis B and human papillomavirus infection (HPV), improve the early diagnosis of malignant diseases, increase the education of populations, reinforce technical and medication resources and train health professionals by strengthening multidisciplinary oncology teams.

The delegates earned two European Credit Transfer and Accumulation System credits recognised by the Fernando Pessoa University, Porto Portugal. The theme of the conference was ‘Training to better care’.

## Key conference themes

The key themes identified for the conference were as follows:

1. Instruments for proficient cancer control in PALOP,
2. Oncology education and training in PALOP,
3. CanScreen5—IARC Platform to improve the quality in tracking cancer,
4. IGCS—Global Curriculum & Mentorship Programme,
5. Oncology Training/Intervention—Support programmes,
6. Telepathology and Cancer: Challenges and Opportunities. The experience of Mozambique,
7. Cancer Burden in the PALOP region and Sub-Saharan Africa,
8. Breast Cancer—The current situation in PALOP,
9. What is the primary level of cancer care?
10. The African Breast Cancer—Disparities in outcomes study in PALOP Countries (ABC-DO-PALOP) study: A proposal.

## Instruments for proficient cancer control in PALOP

Throughout the conference, the importance of the instruments for proficient cancer control was stressed. For example, the national cancer control programme is a crucial instrument, given that it is a public health programme that indicates the actions needed to reduce the number of cancer cases, increase survival and improve the quality of life of cancer patients [3]. In this context, the main points of the Cape Verde cancer control plan were presented by Dr Carla Barbosa, which follows the WHO recommendations and includes the following points: prevention, early detection, diagnosis and treatment, palliative care, policy and advocacy. Data from the two population-based cancer registries, namely Beira and Maputo in Mozambique, were presented by Prof. Cesaltina Lorenzoni [4]. The importance of organisational aspects was discussed and presented to the African Cancer Registry Network as the institution that supports the organisation of population-based registries in Africa. The Angolan Institute for Cancer Control was also presented by Dr Isabel Candido, as an example of a hospital unit solely dedicated to oncology, which aims to become a comprehensive cancer centre in Angola [2]. The participants interacted, and the need for these three instruments was stressed: the national cancer control plan, the population-based cancer registry and the cancer units for the fight against cancer to be effective. The difficulties and successes encountered by the PALOP were highlighted.

## Oncology education and training in PALOP

An ongoing issue for cancer control is the training of specialists in oncology. This panel discussed the training programmes in surgical oncology, medical oncology and radio-oncology, which has been approved in Angola, Cape Verde and Mozambique. The training experiences were presented, and the need for training recognised by the PALOP countries that have scientific support from AORTIC, as well as by the colleges of medical specialties of the Medical Association, was emphasised for each country. The fact that there is a common official language makes this task easier. A joint oncology school for PALOP would be an instrument of education in fundamental oncology. It was decided to lay the foundation for this to happen and to be able to conduct quality e-learning in oncology whilst taking into account the situation of the pandemic and financial constraints The expertise that is already being received in both Cabo Verde and Mozambique, sponsored by ECHO and the Calouste Gulbenkian Foundation, is an example [2]. The need for internships in comprehensive cancer centres abroad and a strong training component according to local realities was offered as a suggestion. The speakers were Dr Herondina B Spencer (medical oncology), Dr Nilton C Rosa (surgical oncology) and Dr Gudo Morais (radio-oncology).

## CanScreen5—IARC platform to improve quality in tracking cancer

The Cancer Screening in Five Continents (CanScreen5) initiated by the International Agency for Research on Cancer (IARC) is a global repository of information on cancer screening programmes, which aims to collect, analyse and disseminate information on the characteristics and performance of cancer screening programmes in various countries in a harmonised manner and on a regular basis. The data forms were designed to collect only uniform and standardised aggregated data without any personal or sensitive information. No individual records are received or stored in the CanScreen5 database platform, to avoid any privacy or confidentiality breach or concerns. The core objective of CanScreen5 is to encourage and support countries to use cancer screening data for programme evaluation and quality improvement. A web-based open-access platform (http://canscreen5.iarc.fr/) was designed to facilitate an access to and interpretation of data from the cancer screening programmes worldwide [5]. The initiative will also motivate and support programme managers to improve health information systems and evaluate cancer screening programmes for the better overall management, quality assurance and informed policy-making. Among those, the capacity building by training for better programme evaluation and quality improvement is the major focus of the CanScreen5 global network. A few tailored training programmes are ongoing: one designed to tackle social inequalities on cancer screening in Latin America and the Caribbean and the other to better understand on-going screening activities in Sub-Saharan Africa. This initiative is expected to have a positive impact on the planning and quality of cancer screening programmes around the world.

## IGCS—Global Curriculum and Mentorship Programme

Cervical cancer ranks as the fourth most commonly diagnosed cancer among women worldwide. More than 85% of cervical cancer cases occur in low- and middle-income countries, where there are not enough medical specialists to provide quality screening, diagnostic and treatment services. Treatment equipment and services are lacking, and the shortages of infrastructure and pathology services are common [6–8]. In several low- and middle-income countries (LMICs), there are no gynaecologic oncologists nor a lack of basic surgical care. The IGCS provides a Gynaecologic Oncology Global Curriculum and Mentorship Programme, a comprehensive 2-year education and training programme to train gynaecologists in gynaecologic oncology in countries with no formal training programme in this speciality [9]. According to Prof. Mila Pontremoli Salcedo from Universidade Federal de Ciências da Saúde de Porto Alegre, Brazil, and MD Anderson Cancer Centre, USA, Mozambique was one of the pilot sites for the IGCS Global Curriculum and initiated the programme in 2017 [9, 10]. In Mozambique, there are three fellows participating in the programme. They are expected to graduate in 2020. In Mozambique, cervical cancer prevention courses are provided [11]. The courses have transitioned from international faculty only to now include Mozambican gynaecologists as lecturers and trainer, including two of the fellows from the IGCS global curriculum programme. The fellows are now faculty for cervical cancer prevention courses, delivering lectures, supervising skills training stations and serving as gynaecologic oncology and cancer prevention referral physicians for local providers. Dr Ricardina Rangeiro reiterated that the IGCS programme allows onco-gynaecologists to be trained in places that lack expertise. The fact that learning takes place in hospitals and with the resources that they have available makes this programme even more compelling. It is a privilege to learn from such knowledgeable mentors and to be able to treat patients with the know-how acquired.

## Oncology training/intervention—support programmes

Prof. Ellen Baker is the director of Project Extension for Community Healthcare Outcomes (ECHO®), a teleconsulting and telementoring partnership between MD Anderson specialists and providers in rural and underserved communities including African countries. The mission of this project, its principles and the results of its intervention in Mozambique were also described. The training of health professionals, including practical training, development of accessible technologies for the prevention, diagnosis and treatment of cancer and strengthening the health system through partnerships, has been the main focus. The ECHO project develops actions towards the prevention of cervical cancer in Mozambique and the creation of a team of oncological gynaecologists. This project also contributed to the national cancer control plan of Mozambique [12].

Prof. João Almeida Pedro, from the Calouste Gulbenkian Foundation, addressed the project on improving the diagnosis and treatment of oncological diseases in Cape Verde and Mozambique. This project aims to improve the performance and quality of the services provided and their adequacy to the needs of the population. The modus operandis is specialised training in Portugal, using internships in a clinical context of variable duration and focussing in oncology, ‘On the job’ training and technical assistance locally, acquisition of specialised clinical equipment and promotion of research and academic training. The Gulbenkian Foundation has been providing support for Mozambican and Cape Verdian medical and non-medical staff to be trained in Portugal in various cancer specialties (surgery, radiation therapy, medical oncology, oncological nursing and pathology, among others) during short-term fellowships, allowing for an effective education in multidisciplinary cancer care. This has led to the implementation of new surgical cancer techniques and multidisciplinary tumour boards in Mozambique and Cape Verde [13].

## Telepathology and cancer: challenges and opportunities: the experience of Mozambique

Telepathology provides a versatile tool to improve clinical care and foster the educational and research opportunities in Africa, whose countries have a critical shortage of pathologists and have a strong need for the improvement of healthcare infrastructure and adequate personnel training. The pathology department of the Maputo Central Hospital, Mozambique, has developed a regular telepathology programme since 2009 with the support of pathologists at Hospital Clinic of Barcelona, Spain. This programme consists of training of pathologists, residents and diagnostic teleconsultation, using initially digital video microscope system, and more recently, a whole slide imaging system. Since 2015, the same system is used for undergraduate education in pathology (interactive digital microscopy) in a collaborative project between the Departments of Pathology of Eduardo Mondlane and Porto Universities.

In June 2020, Angola, Cape Verde and Mozambique joined in a similar activity through a Pathology Project ECHO Lusophone Africa, together with the Memorial Sloan-Kettering Cancer Centre and the Brazilian Society of Pathology. This includes a monthly session for a short didactic lecture and case discussion. These examples demonstrate that telepathology is feasible in the resource-limited settings. Its use gives support and can improve pathological diagnosis, clinical care and education. Prof. Carla Carrilho from Mozambique co-ordinated this session.

## Cancer burden in the PALOP region and Sub-Saharan Africa

The burden of cancer is increasing in Africa in general and in the PALOP countries, in particular, because of the aging and growth of the population as well as an increased prevalence of risk factors associated with economic transition, including smoking, obesity, physical inactivity and reproductive behaviours. The most prevalent malignant tumours continue to be cervical, breast, haematological, prostate and digestive tumours. Many are preventable, and some are associated with infections. Paediatric malignant tumours are also a growing health problem [14, 15]. During the conference session, it was possible to discuss the history and profile of these malignancies and the prevention and treatment with African experts. Recommendations, results of clinical trials and new therapeutic options were also discussed. Current and future research collaboration was another area that was addressed. The speakers were Dr Christopher Williams, Prof. Nicholas Abinya, Dr Sitna Mwanzi, Prof. Abubakar Bello, Dr Carlos Selemane, Dr Paulo Salamanca, Dra Noémia Afonso and Dr Amad Faizana.

## Breast cancer—the current situation in PALOP

A special session on breast cancer was organised with the support of ROCHE, which aimed to clarify the situation in Portuguese-speaking countries and to address new therapies approved internationally. Breast cancer (BC) is the second most common cancer among women in PALOP countries. The incidence rate is clearly increasing [2, 4, 16]. In Sub-Saharan Africa countries, BC clinical presentation differs from that in high-income countries mostly due to late-stage disease at presentation, high proportion of young patients (as the African population is predominantly young) as well as very high mortality rates [17]. A similar picture was presented by pathologists from Angola, Cape Verde and Mozambique [4, 18, 19, 20].

Prof. Carlos Silva Lopes, who studied 373 breast carcinomas diagnosed consecutively in Angola, verified that the molecular profile (Like) was Luminal A—23.8%, Luminal B—27.1%, Luminal B/HER2—8.2%, HER2—14.7% and triple negative—26.2%.

In Mozambique, breast cancer is the third most frequent cancer in women, responsible for 9.5% of the cases [1]. Data from the population-based registries of Maputo and Beira cities show an age-standardised incidence of 15.5 and 14.1, respectively [21]. Although relative risk was lower than other African regions, the incidence of breast cancer in 2015–2016 increased five-fold in Maputo city when compared to the registry from 1956 to 1961 [4]. Data were collected from 262 patients of a cohort prospective study performed in 2015–2017 [22], more than 80% were of the NST histologic type and 72% were diagnosed in late-stage III and IV. The most frequent subtype was Luminal B like, and nearly half of the patients have HER2+ tumours or triple-negative breast cancer (TNBC). Survival was poor, especially among patients with HER2+ and TNBC. In this session, the importance of pathology was emphasised, as well as the quality of the pre-analytical and post-analytical phases for good-quality diagnosis and reporting.

## Health system strengthening is the paramount relevance to improve cancer care in low- and middle-income countries: what is the primary level of cancer care?

Dr Blasques de Oliveira, expert in Comprehensive Primary Care, addressed this topic related to oncology. According to Dr de Oliveira, health systems are open complex systems that respond to small influences with significant responses that challenge the system and originate turbulence and the need for new solutions.

This complexity is clearly seen in the growing double burden of infectious and non-communicable diseases that LMIC are facing.

Cancer along with hypertension and diabetes is gaining more prominence in the morbidity and mortality burden of these countries and along with injuries stresses the already weak health systems within these countries.

Even if the majority of the burden of disease is still linked to infectious diseases, it is possible to identify areas, where synergetic health prevention and promotion interventions at the primary care level can be conducted with impact, both in infectious diseases and non-communicable diseases, including cancer. HPV vaccination and mass control of schistosomiasis are some examples.

Furthermore, common causes linked to lifestyles for hypertension, diabetes and cancer can be tackled by IEC/BCC interventions at primary care level. However, it is also important that more specialised services to be available even at municipal level to provide capacity within hospitals to have at least surgical services and progressively being able to provide diagnosis, follow-up treatment and palliative care to cancer patients.

Training of health professionals, nurses and doctors—primary care, internal medicine, paediatricians and obstetrics and gynaecology—on these diseases and their causes must be conducted, urgently. General surgeons must be informed and trained on the basics of cancer management, to serve in Provincial Hospitals and progressively in intermunicipal and municipal hospitals that have significant case loads.

Better understanding and knowledge on cancer diagnosis and management, reporting and cancer registry capacity, must be decentralised, initially to provincial-level hospitals, and thereafter, this level would have the capacity to provide referrals to municipal (district) levels.

Dr de Oliveira predicts that for a long time to come that provincial hospitals will also be the ‘primary level’ for cancer care with an expanding network of municipal hospitals that will be functionally linked to them, on a systematic vision for better health outcomes.

## The ABC-DO-PALOP study: a proposal

It is estimated that breast cancer mortality will increase about twice in Sub-Saharan Africa (SSA) by 2040. On the other hand, low survival rates for this potentially curable cancer are observed in this region of the world. Thus, it is necessary to create the fundamental conditions in order to improve treatment, follow-up and survival rates. According to Isabel Santos-Silva, Professor of Epidemiology at the London School of Hygiene and Tropical Medicine, United Kingdom, the African Breast Cancer—Disparities in Outcomes (ABC-DO), funded by the US-based Susan G. Komen, was launched in 2014, jointly by the International Agency for Research on Cancer (V McCormack), the London School of Hygiene & Tropical Medicine (I dos-Santos-Silva) and institutions in five SSA countries, namely Namibia, Nigeria, South Africa, Uganda and Zambia [23]. This prospective multi-country study—the largest survival cohort in SSA with over 2100 breast cancer women enrolled at 8 hospitals from diverse healthcare systems with different oncology services—has collected rich demographic, socio-economic, cultural, breast cancer awareness data [24], delays in diagnosis [25], clinical [26], therapeutic [27] and molecular data as well as data on both survival [28] and survivorship outcomes. The follow-up of this cohort will continue until the end of 2022 (funded by a recently awarded grant from the US National Cancer Institute) when all participants will have reached the standard 5+ years follow-up and will enable in-depth investigations of 5-year survival and survivorship. The unique features of the ABC-DO study include: (i) potential transferability of methodology and study instruments in other SSA settings to allow various centres to join the study at a later stage, (ii) focus on between-setting comparisons across SSA to provide more realistic targets for breast cancer control in the region then conduct comparisons with high-income countries, (iii) standardised data collection across settings from all newly diagnosed breast cancer patients seen in each centre over a 2-year period, (iv) prospective follow-up based on frequent contacts (every 3 months) for at least 5 years to minimise losses [29] and (iv) use of m-Health technologies to maximise follow-up, ensure high-quality data collection, allow remote access to the data in real-time for monitoring and provide contemporaneous up-to-date survival estimates (by avoiding the usual long delays in data collection and reporting that tend to affect cancer registries). During the IV Congress PALOP AORTIC, a proposal was presented to extend the ABC-DO study to three Portuguese-speaking African studies (PALOP): Angola, Cabo Verde and Mozambique. Breast cancer is the first or second most common cancer in women in these countries, but few data are currently available on which to draw the effective strategies to control this disease.

## Recommendations

The PALOP Oncology Group met from the 29th to the 31st of July 2020 at the IV PALOP-AORTIC virtual congress in Angola and proposed the following recommendations:

- Strengthen your organisation by creating accessible and useful computer communication channels.
- Create a PALOP Oncology Group website.
- This website will include various working groups: pathology, surgical oncology, clinical/medical oncology, radio-oncology, nursing oncology pharmacy and psycho-oncology. Where justified, further working groups will be added;
- The website will be a collection of the PALOP School of Oncology teaching resources. It will be a place to support formal and informal oncology education. It will also accommodate all works presented in the form of a poster or articles (without prejudice to property rights);
- The website will also be the place to promote work or scientific meetings similar to the PALOP IV that has just taken place. For this, resources for virtual meetings will be developed.
- Undertake to make efforts to integrate Guinea-Bissau and São Tomé and Principé as soon as possible.
- The PALOP oncology group urges all its members to become the members of AORTIC and to participate actively in all its activities and working groups and to further strengthen their relations.
- The PALOP Oncology Group considers it important to harmonise training programmes, treatment recommendations for oncological diseases and calls for the efforts of the Health Services to continue to provide resources for oncology services in the various countries.
- Gratitude and appeals to the Gulbenkian Foundation and the ECHO/IGCS Programme to extend all interventions as far as possible to all PALOP.
- The PALOP Group of Oncology considers it important to contact and strengthen collaboration with similar organisations of the CPLP and with IARC.
- It will make every effort to secure oncological drug and technical support to PALOP countries.
- It will create its statutes in a democratic and representative way and thanks to the Angolan Ministry of Health and Roche for the commitment and assistance in this historic conference, the first held virtually.

## Conflicts of interest

The authors declare that they have no conflicts of interest.

## Funding

No specific funding was received for the publication of this article

## Authors’ contributions

LLS participated in the design, coordinated all contributions and drafted the manuscript. LLS, HBS, FM, ST, BR, LVL and HF performed critical revisions for important intellectual content of the article. LVL helped with the synthesis. BR corrected the final manuscript. All authors read and approved the final manuscript.

## Ethics approval and consent to participate

The organisation committee of the PALOP cancer meeting in Angola and its ethics office authorised the publication of this manuscript.

## Consent for publication

During the event, all the participants authorised the taking of photos, video and their publication at the time of registration in accordance with the current privacy protection policy. The publication of the remaining photographs has been authorised by the official services that are the owners of these photos, fulfilling what is established internally in its policies of protection and privacy.
